# Mapping the protein binding site of the (pro)renin receptor using in silico 3D structural analysis

**DOI:** 10.1038/s41440-022-01094-w

**Published:** 2022-12-09

**Authors:** Akio Ebihara, Daiki Sugihara, Makoto Matsuyama, Chiharu Suzuki-Nakagawa, A. H. M. Nurun Nabi, Tsutomu Nakagawa, Akira Nishiyama, Fumiaki Suzuki

**Affiliations:** 1 Faculty of Applied Biological Sciences, Gifu University, Tokai National Higher Education and Research System, 1-1 Yanagido, Gifu, 501-1193 Japan; 2 Center for Highly Advanced Integration of Nano and Life Sciences (G-CHAIN), Gifu University, Tokai National Higher Education and Research System, 1-1 Yanagido, Gifu, 501-1193 Japan; 3 Preemptive Food Research Center (PFRC), Gifu University Institute for Advanced Study, 1-1 Yanagido, Gifu, 501-1193 Japan; 4 Department of Chemical Engineering, Indian Institute of Technology Guwahati, Guwahati, Assam 781039 India; 5 Graduate School of Natural Science and Technology, Gifu University, Tokai National Higher Education and Research System, 1-1 Yanagido, Gifu, 501-1193 Japan; 6 Division of Molecular Genetics, Shigei Medical Research Institute, Okayama, Minami 701-0202 Japan; 7 Laboratory of Population Genetics, Department of Biochemistry and Molecular Biology, University of Dhaka, Dhaka, 1000 Bangladesh; 8 Department of Pharmacology, Faculty of Medicine, Kagawa University, Miki, Kagawa 761-0793 Japan

**Keywords:** (Pro)renin receptor, Pancreatic ductal adenocarcinoma, AlphaFold2, Intrinsically disordered region, Wnt/β-catenin signaling

## Abstract

We have previously reported that monoclonal antibodies against the (pro)renin receptor [(P)RR] can reduce the Wnt/β-catenin-dependent development of pancreatic ductal adenocarcinoma (PDAC), the most common pancreatic cancer. Antibodies against two (P)RR regions (residues 47–60 and 200–213) located in the extracellular domain (ECD) reduced the proliferation of human PDAC cells in vitro. Although these regions probably participate in the activation of Wnt/β-catenin signaling, their functional significance remains unclear. Moreover, the (P)RR ECD is predicted to possess an intrinsically disordered region (IDR), which allows multiple protein interactions because of its conformational flexibility. In this study, we investigated the significance of the two regions and the IDR by in silico 3D structural analysis using the AlphaFold2 program and evolutionary sequence conservation profile. The model showed that ECD adopted a folded domain (residues 17–269) and had an IDR (residues 270–296). The two regions mapped onto the structural model formed a continuous surface patch comprising evolutionarily conserved hydrophobic residues. The homodimeric structure predicted by AlphaFold2 showed that full-length (P)RR comprising the ECD, single-span transmembrane, and cytoplasmic domains formed a twofold symmetric dimer via the ECD, which explains the experimentally proven homodimerization. The dimer model possessed two hand-shaped grooves with residues 47–60 and 200–213 in their palms and the IDR as their fingers. Based on these findings, we propose that the IDR-containing hydrophobic grooves act as a binding site for (P)RR and perform multiple functions, including Wnt signaling activation.

**Figure:**
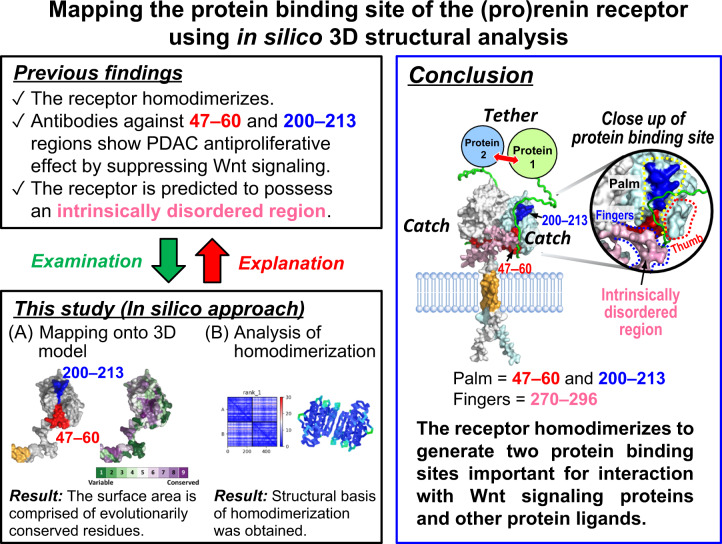
Antibodies against the (pro)renin receptor residues 47–60 and 200–213 can inhibit pancreatic ductal adenocarcinoma (PDAC) cell proliferation by suppressing Wnt signaling. This study provides 3D structural insights into receptor binding and one-to-many interactions, which underpin the functional versatility of this receptor.

Antibodies against the (pro)renin receptor residues 47–60 and 200–213 can inhibit pancreatic ductal adenocarcinoma (PDAC) cell proliferation by suppressing Wnt signaling. This study provides 3D structural insights into receptor binding and one-to-many interactions, which underpin the functional versatility of this receptor.

## Abstract (web summary)

Antibodies against the (pro)renin receptor residues 47–60 and 200–213 can inhibit pancreatic ductal adenocarcinoma (PDAC) cell proliferation by suppressing Wnt signaling. This study provides 3D structural insights into receptor binding and one-to-many interactions, which underpin the functional versatility of this receptor.

## Introduction

The (pro)renin receptor [(P)RR] is a single-span transmembrane protein originally identified as a regulator of the renin–angiotensin system (RAS) required to maintain blood pressure and body fluid balance [1]. (P)RR reportedly contributes to the pathogenesis of various diseases, including fibrosis, hypertension, preeclampsia, diabetic microangiopathy, and cancer [2]. In particular, the aberrant expression of (P)RR directly leads to genomic instability in human pancreatic ductal epithelial cells and contributes to the early carcinogenesis of pancreatic ductal adenocarcinoma (PDAC) [3].

An open question regarding (P)RR functionality is its one-to-many binding; specifically, (P)RR interacts with various RAS-independent binding partners [2, 4, 5]. Receptor binding is based mainly on interactions between the extracellular domain (ECD) and the respective signaling protein ligands. For example, prorenin acts as a (P)RR ligand in the RAS-dependent pathway [1, 5]. (P)RR binding is mediated by the ECD, leading to the nonproteolytic activation of prorenin [6, 7] and the activation of local tissue RAS [2]. This binding not only induces intracellular tyrosine phosphorylation-dependent signaling pathways [1, 2] but also leads to the downregulation of (P)RR and upregulation of phosphatidylinositol-3 kinase by binding of the transcription factor promyelocytic zinc finger protein with the (P)RR cytoplasmic domain [8]. (P)RR undergoes intracellular processing to produce three different forms: a full-length form [1], a truncated membrane-bound form [9], and a truncated soluble form lacking the transmembrane domain, termed soluble (P)RR [s(P)RR] [10]. The truncated membrane-bound form remains inside the cell and interacts with vacuolar H^+^-ATPase (V-ATPase) as a component of the multisubunit membrane-bound proton pump [2, 9]. s(P)RR, which comprises most of the ECD, is secreted extracellularly and interacts with Frizzled-8 (FZD8) on the surface of the renal collecting duct principal cells, enhancing the urine-concentrating capability via FZD8-dependent β-catenin signaling [11, 12]. The full-length (P)RR is a component of the Wnt receptor complex [13]. The (P)RR ECD is required for binding to FZD8 and low-density lipoprotein receptor-related protein 6 (LRP6) to maintain Wnt/β-catenin signaling [13]. (P)RR also functions in a RAS-independent manner as an adaptor between the Wnt receptor complex and V-ATPase, which allows the acidification of Wnt signalosome vesicles and subsequent LRP6 phosphorylation [13]. Furthermore, (P)RR interacts with partitioning defective 3 homolog (laminar formation) and pyruvate dehydrogenase subunit (energy metabolism) [2, 4, 5]. Thus, (P)RR could potentially perform multiple modulatory functions by interacting with various proteins, but these interacting partners do not share significant similarities in protein sequence.

The silencing of (P)RR suppresses Wnt signaling activation, thus reducing the proliferative activity of human PDAC cells and the growth of engrafted tumors in nude mice [14]. Monoclonal antibodies (mAbs) against residues 200–213 located in the (P)RR ECD reduce PDAC cell proliferation in vitro as well as PDAC tumor growth in vivo by suppressing the activation of Wnt signaling [15]. Before generating the neutralizing mAbs, we examined the antiproliferative effects on PDAC cell growth of seven antipeptide polyclonal antibodies (pAbs) against the (P)RR ECD [15]. pAbs against two regions (residues 47–60 and 200–213) significantly reduced cell proliferation in a dose-dependent manner [15]. Although these regions probably participate in the activation of Wnt/β-catenin signaling, their functional significance remains unclear.

We previously suggested that residues 269–292 located in the (P)RR ECD form an intrinsically disordered region (IDR) [16]. IDRs adopt various conformations under physiological conditions [17] and can utilize the same sequence region in the sequence to bind multiple partners. In addition, IDR-equipped proteins can function as hubs in protein–protein interaction networks, which are essential for cell signaling [18, 19]. A reasonable expectation is that the (P)RR IDR contributes to the multiple binding and functions of this receptor. Nonetheless, the functionality of the (P)RR IDR has not yet been reported.

The 3D structures of mammalian V-ATPase have been determined by cryo-electron microscopy (cryo-EM) and show that the truncated membrane-bound form of (P)RR binds to the inside of the V-ATPase c-ring [20, 21]. Although mass spectrometry analysis has detected some full-length (P)RR in protein preparation [20], the (P)RR ECD is missing in these cryo-EM structures, and the truncated membrane-bound form alone is visible [20, 21]. To date, the 3D structure of the (P)RR ECD has not been experimentally determined. The full-length (P)RR structure has been predicted using a threading-based program [22]. Recently, significant progress in protein 3D structure prediction has been made using AlphaFold2 (AF2) [23] and RoseTTAFold [24], in which a protein 3D structural model is generated using machine learning algorithms with amino acid sequences as the only input. Both programs can predict protein structures with near-experimental accuracy [23, 24]. Notably, the AF2 program has predicted protein 3D structures on the human proteome scale [25], and a structural model of human (P)RR is available in the AlphaFold Protein Structure Database [26]. Therefore, it would be intriguing to utilize a 3D structural model to provide a novel perspective on (P)RR functions. In this study, we analyzed the 3D structure of (P)RR in silico using the AF2 program and evolutionary sequence conservation profile, and we investigated the functional significance of the two regions involved in the PDAC antiproliferative effect and (P)RR IDR.

## Methods

### Data source

The Protein Data Bank (PDB) coordinate file of human (P)RR was downloaded from the AlphaFold Protein Structure Database [26] with accession ID AF-O75787-F1. Predicted local-distance difference test (pLDDT) scores for each residue were stored in the B-factor field of the downloaded coordinate file. The average pLDDT score of the structural model was calculated using the WHAT IF web server [27]. The positions of the domain boundaries were referenced according to the UniProt database [28]. The ProSA Z score was obtained using a web server [29]. The Ramachandran plot is available from PDBsum [30].

The PDB coordinate files of FZD8 and LRP6 are available in the database [26] as accession IDs FZD8, AF-Q9H461-F1 and LRP6, AF-O75581-F1.

### Structure prediction of human (P)RR with RoseTTAFold

A structural model of human (P)RR (residues 1–350) was predicted using the RoseTTAFold Google Colab Notebook [24, 31] with the mmseqs2 multiple sequence alignment (MSA) method without templates. The pLDDT scores for each residue were stored in the B-factor field of the resulting coordinate file. PyMOL [32] was used to superimpose residues 17–269 of the resulting RoseTTAFold model on the AF2 model and to calculate the root mean square deviation (RMSD) values between the models.

### Structural similarity analysis of human (P)RR

The Dali server [33] was used to identify proteins structurally similar to human (P)RR. The human (P)RR AF2 model (accession ID: AF-O75787-F1) was used as the query protein structure against the PDB25 database [33]. The resulting Dali output coordinate files were superimposed on the human (P)RR structure and visualized using PyMOL.

### Evolutionary conservation analysis of human (P)RR

The ConSurf web server [34] was used to estimate evolutionary sequence conservation, and the human (P)RR AF2 model (accession ID: AF-O75787-F1) was submitted to the server (https://consurf.tau.ac.il/). Homologous sequences were identified using the HMMER algorithm against the UniRef-90 protein database. The Bayesian calculation method was used to calculate position-specific conservation scores. The resulting ConSurf scores assigned to each (P)RR amino acid residue were stored in the B-factor field of the PDB file. Homologous sequences were aligned using Clustal Omega [35]. The alignment figure was generated using ESPript [36]. GraphPad Prism 9.3 (GraphPad Software, La Jolla, CA, USA) was used to calculate the Consurf scores of the (P)RR residues.

### Analysis of homodimerization of human (P)RR

AlphaFold2_advanced Notebook [31] was used to obtain a structural model for the two chains of the (P)RR ECD (residues 17–270) or two chains of full-length (P)RR (residues 17–350). The model building parameters were as follows: MSA_method, mmseqs2; homooligomer, 2; use_templates, false; default for other parameters. The resulting PDB coordinate files, 3D structural models colored with the pLDDT score, and predicted aligned error (PAE) plots were used to examine homodimer formation. The ProSA Z score was obtained using a web server [29]. A Ramachandran plot was generated using PDBsum [30]. The solvent accessibility and dimerization interface were analyzed using the PDBePISA server [37].

### Molecular graphics analysis

Molecular images were produced using PyMOL [32]. To map the conservation profile onto the 3D structural model, a PDB file with the ConSurf scores was used to produce a surface representation. The electrostatic potential was calculated using the APBS algorithm in the PyMOL plugin, and the surface representation was colored according to the normalized consensus hydrophobicity scale [38] to identify hydrophobic surface patches.

## Results

### Quality of the predicted human (P)RR model

**Fig. 1:**
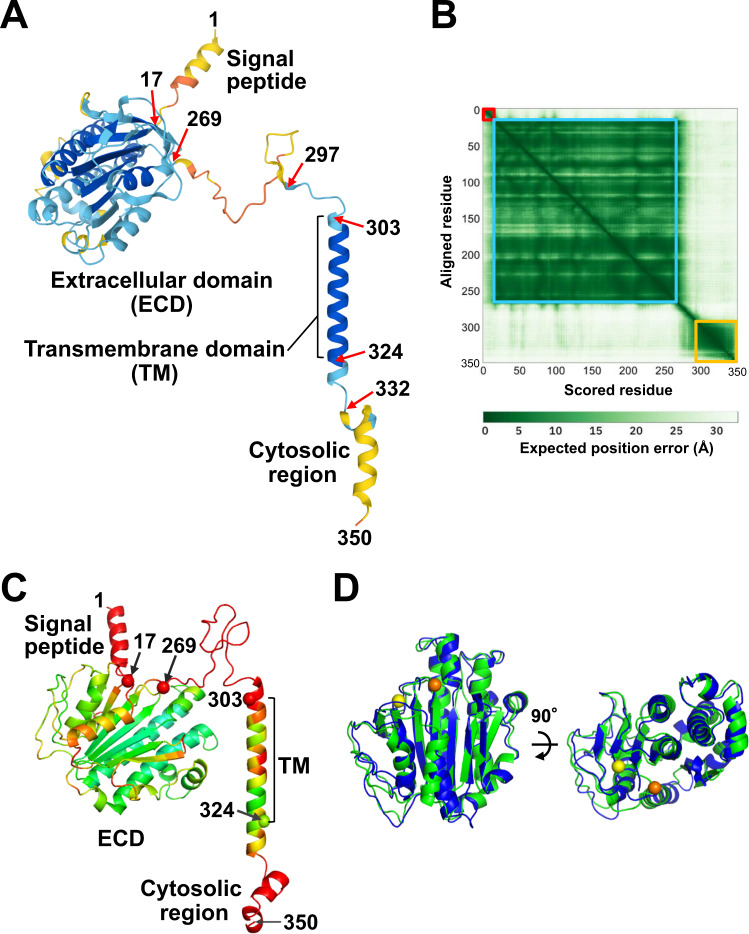
Predicted structure of human (P)RR. **A** Cartoon representation of the AF2 structural model. The model includes the signal peptide (residues 1–16), extracellular domain (ECD; residues 17–302), transmembrane domain (TM; residues 303–323), and cytosolic region (residues 324–350). The structure is colored according to the predicted local-distance difference test (pLDDT) score in blue, cyan, yellow, and orange for the regions that were most confidently predicted (pLDDT > 90), confidently predicted (90 > pLDDT >70), predicted with low confidence (70 > pLDDT >50), and predicted with very low confidence (pLDDT < 50), respectively. **B** Predicted aligned error (PAE) plot. The PAE values of residues 1–16, 17–269, and 290–350 are illustrated with overlaid squares in red, cyan, and yellow, respectively. **C** Cartoon representation of the RoseTTAFold structural model. The structure is colored according to the pLDDT: blue for the most confidently predicted regions (pLDDT ≥ 0.9) and red for those predicted with very low confidence (pLDDT ≤ 0.5) based on a color spectrum of red, yellow, green, cyan, and blue. **D** Secondary structure-based superimposition of RoseTTAFold ECD (green) on AF2 ECD (blue). Cα atoms of residues 17 and 269 of the RoseTTAFold model are shown as yellow and orange spheres, respectively. The right view is the same representation rotated by 90°

The AF2 structural model of human (P)RR is shown in Fig. 1A. The model quality was assessed using the ProSA Z score, Ramachandran plot, and pLDDT score. The ProSA Z score is indicative of the overall protein structure quality and can be used to check whether the input structure is within the range of scores typically found for native proteins of similar size [29]. The structure model had a ProSA Z score of −5.83, and the model was plotted in the region of the structures obtained by X-ray crystallography (Supplementary Fig. 1A). The Ramachandran plot showed that 88.5% of the residues were situated in the most favored regions, and the remaining 11.5% were in the additional allowed regions (Supplementary Fig. 1B). The pLDDT score is a per-residue prediction confidence metric expressed on a scale from 0 to 100 using the AF2 program [23]. The average pLDDT score of the human (P)RR AF2 model was 81.3, indicating that the model was predicted confidently (pLDDT > 70).

Both the folded the ECD (residues 17–269) and the transmembrane domain (TM)-containing region (residues 297–332) were predicted with high confidence (Fig. 1A). In contrast, the signal peptide, residues 270–296, and cytoplasmic domain were modeled with low confidence (Fig. 1A). The PAE plot of human (P)RR indicated high confidence in the relative position and orientation of the domains at residues 1–16, 17–269, and 290–350 (Fig. 1B). Moreover, the plot suggested an interdomain movement between the two domains involving the intervening residues 270–289. Residues 269–292 were predicted to form an IDR using sequence-based disorder prediction [16]. Approximately 30% of human protein residues are predicted with low confidence (pLDDT < 70) using AF2; [25, 39] low-confidence residues are proposed to encompass both IDRs and regions that are structured upon complex formation [25]. Hence, residues 270–296, which had low pLDDT scores, probably formed an IDR.

To validate the model reliability, a structural model of human (P)RR (residues 1–350) was predicted using the RoseTTAFold program [24, 31] (Fig. 1C), and the average pLDDT score was 0.598 on a scale of 0–1.0. The TM-containing region and the folded domain of the ECD were modeled with high confidence, whereas the signal peptide, residues 269–303, and the cytosolic region were modeled with low confidence (Fig. 1C). In the cryo-EM structure [20], the truncated membrane-bound (P)RR (residues 292–343) consists of a long α-helix and a short α-helical turn connected by an extended linker. The conformations of these (P)RR residues modeled by AF2 (Fig. 1A) and RoseTTAFold (Fig. 1C) were similar to those in the cryo-EM structure [20]. The RoseTTAFold ECD and AF2 ECD models were well superimposed, with a root mean square deviation (RMSD) value of 1.3 Å for their Cα atoms (Fig. 1D). Although both prediction programs provided an essentially similar structural model of human (P)RR ECD, we used AF2 prediction because modeling monomers and multimeric protein complexes is feasible using the user-friendly AF2 prediction platform [31].

### (P)RR and alkaline phosphatase (ALP) structural similarities

**Fig. 2:**
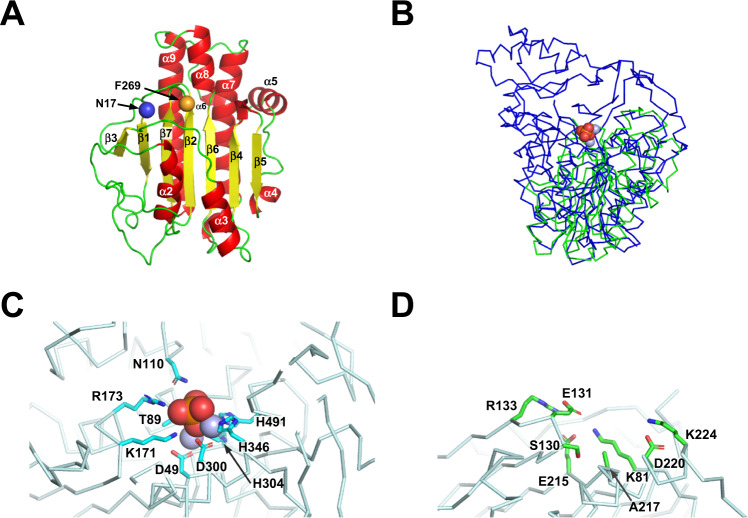
Structural similarity of human (P)RR to alkaline phosphatase (ALP) family proteins. **A** The overall structure of the ECD with a cartoon representation (α-helices shown in red and β-strands in yellow). The Cα atom of residue 17 is shown as a blue sphere, and that of residue 269 is shown as an orange sphere. **B** Structure-based superimposition of human (P)RR ECD (green) on PhoK (blue; PDB ID: 5XWK, residues 31–560). Cα traces of both proteins are shown as ribbons. **C** PhoK active site. Active site residues are shown as sticks. Two bound zinc centers (purple) and a phosphate ion (oxygen atom, red; phosphorus atom, orange) are shown as spheres. **D** The human (P)RR residues that occupy spatially equivalent positions in the ALP active site. Amino acid residues that may bind to a metal and a phosphate ion are shown in stick representation

The folded domain of the human (P)RR ECD (residues 17–269) consists of a seven-stranded mixed β-sheet flanked on both sides by eight α-helices (Fig. 2A). Structural comparisons using the Dali server [33] revealed 36 structurally similar proteins. The top three hits and proteins from mammalian species are listed in Supplementary Table 1. The top match to (P)RR was ALP PhoK. (P)RR ECD and PhoK were superimposed with an RMSD value of 3.7 Å for the Cα atoms of 192 aligned residues (Fig. 2B). Notably, the (P)RR ECD was structurally similar to ALP, phosphodiesterase, and sulfuric ester hydrolase.

The active site of PhoK coordinates two catalytic zinc ions with Asp and His residues, allowing one phosphate ion to bind to the metal ions (Fig. 2C), which is a common active site architecture of ALP [40]. Because (P)RR does not contain the conserved Asp and His residues required to coordinate the catalytic divalent metal(s) (Fig. 2D), (P)RR appears to be a pseudo-ALP.

### Mapping of (P)RR regions involved in PDAC antiproliferative effects

We previously reported that pAbs against two regions (residues 47–60 and 200–213) significantly reduced the proliferation of human PDAC cells in vitro [15]. These regions are likely to play a role in Wnt/β-catenin signaling activation. It is anticipated that functionally important residues are evolutionarily conserved and clustered together to form functional patches in a 3D structure [41, 42]. The functional significance of these two regions in the PDAC antiproliferative effect can be explored by mapping both the residue positions and the evolutionary sequence conservation profile simultaneously on the 3D structure of (P)RR.

**Fig. 3:**
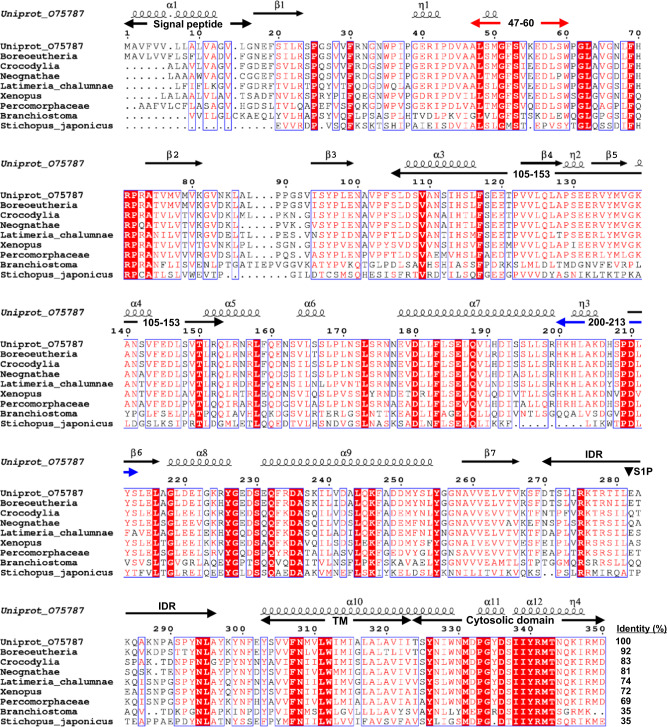
Multiple sequence alignment of human (P)RR and its sequence homologs. The sequence homologs are specified with clade/species names (common name and UniRef cluster ID): *Boreoeutheria* (mammal; UniRef90_P81134), *Crocodylia* (alligator; UniRef90_A0A151NQQ9), *Neognathae* (bird; UniRef90_H0Z8C1), *Latimeria chalumnae* (coelacanth; UniRef90_H3AI83), *Xenopus* (clawed frog, UniRef90_Q5M8F1), *Percomorphaceae* (ray-finned fish; UniRef90_A0A4W6D9M0), *Branchiostoma* (*Amphioxus*; UniRef90_C3YJH1), and *Stichopus japonicus* (sea cucumber; UniRef90_A0A2G8L3G6). Identity (%) represents the percentage sequence identity to human (P)RR. Identical residues are shown as white characters with a red background and similar residues with red characters with a white background. The secondary structures of the human (P)RR AF2 model are shown at the top: α, α-helix; β, β-strand; η, coil. The processing site of site-1 protease (S1P) is depicted as a black triangle

First, we used the ConSurf server [34, 43] to obtain the conservation profile of (P)RR. Using all human (P)RR residues as the input protein sequence, the homolog search algorithm in ConSurf identified 114 nonredundant homologous sequences. The MSA of human (P)RR and eight sequence homologs is presented in Fig. 3. A high level of sequence identity (69–92%) was found among vertebrates, whereas a low sequence identity (35%) was observed among invertebrates distant from humans.

**Fig. 4:**
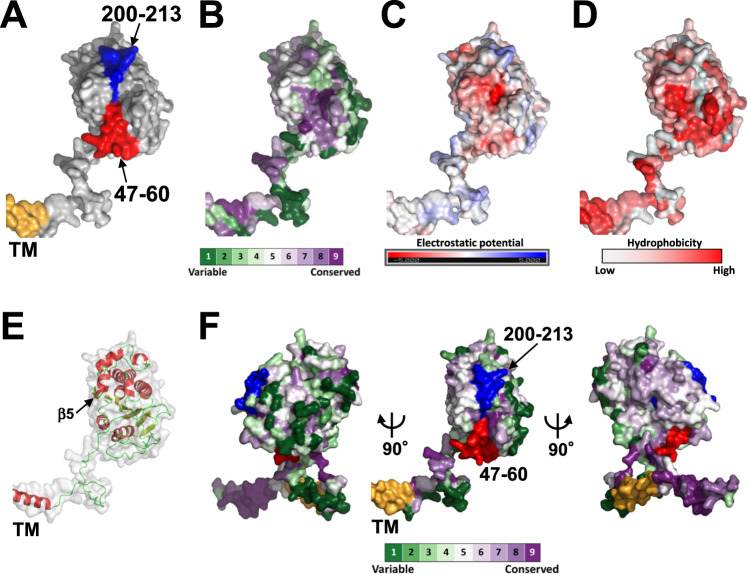
Molecular surface properties of human (P)RR. **A** Surface representation of two regions involved in the antiproliferative effect against PDAC: residues 47–60 (red) and 200–213 (blue). The TM is colored yellow. **B** Evolutionary conservation profile shown in surface representation. Each amino acid residue is colored according to its ConSurf conservation score. The color-coding bar shows the ConSurf coloring scheme, which varies from green (highly variable; score 1) to purple (highly conserved; score 9). **C** Electrostatic surface potential colored from negative (−5 kT/e, red) to positive (+5 kT/e, blue). **D** Surface representation colored by hydrophobicity with a color gradient of red (most hydrophobic) to white (least hydrophobic). **E** Human (P)RR monomer depicted as a gray transparent surface with a cartoon representation (α-helices shown in red and β-strands in yellow). **F** (Middle) Another conservation profile shown in surface representation. Residues 47–60 and 200–213 are shown in red and blue, respectively. (Left and right) Two views are shown of the same representation related by a 90° rotation

Next, we examined the spatial distribution of residues 47–60 and 200–213 using the (P)RR AF2 model. Interestingly, these two regions formed a solvent-accessible continuous patch on the ECD surface (Fig. 4A). When the conservation profile was mapped to the structural model, the surface area around the two regions, especially the 47–60 residue region, consisted of evolutionarily conserved residues (Fig. 4B). Furthermore, the surface area around residues 47–60 had weak electrostatic potential (Fig. 4C) and was hydrophobic (Fig. 4D).

### Structural basis for the homodimerization of (P)RR

Nguyen et al. [1] reported the possibility of the homodimerization of (P)RR. Schefe et al. [8], Contrepas et al. [44] and our group [45] have experimentally demonstrated that (P)RR forms a homodimer, and dimerization occurs via the ECD [45]. The folded domain of the (P)RR ECD constitutes a single β-sheet in the core of the protein flanked by α-helices (Fig. 4E). The domain adopts a semicircular shape with a convex face and a flat face. Interestingly, mapping the conservation profile onto the 3D structural model revealed that the convex shape comprised variable residues (left, Fig. 4F). Simultaneously, the flat face consisted of the conserved residues (right, Fig. 4F). One β-strand (β5) was located on this flat face (Fig. 4E). The participation of an edge strand in homodimerization has been reported previously [46–48]. Thus, we anticipated that the edge β-strands present on the conserved flat surface might align in an antiparallel orientation, forming a unified β-sheet that adopts a homodimeric structure.

**Fig. 5:**
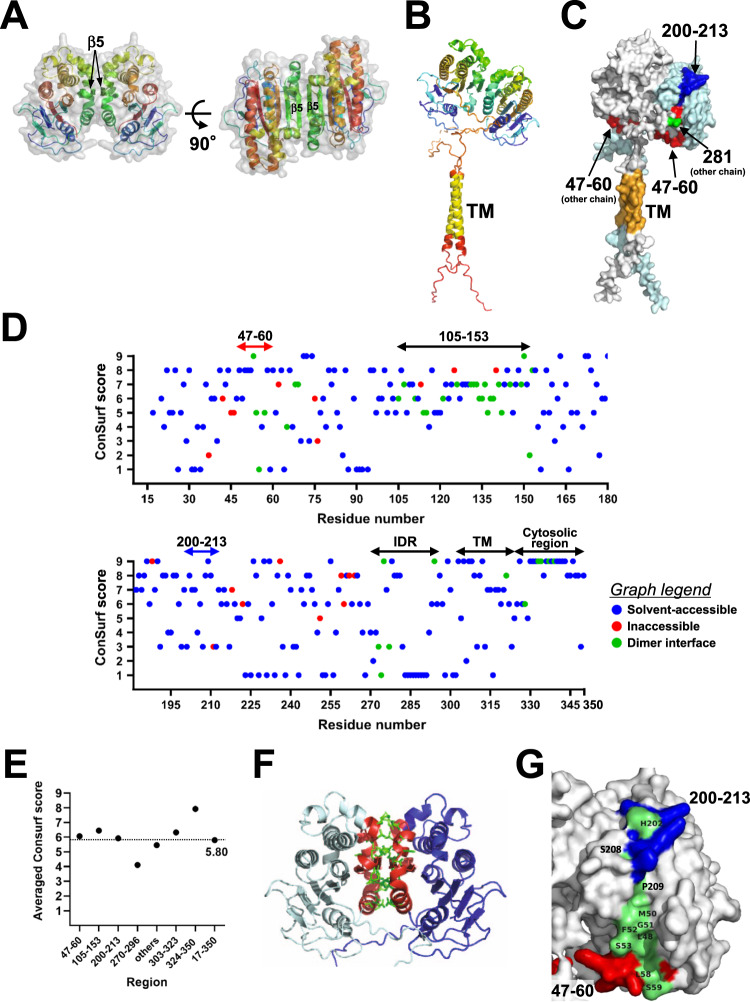
Analysis of predicted homodimeric structures of human (P)RR. **A** Two chains of the ECD (residues 17–270) are depicted as a gray transparent surface with a cartoon representation. Each chain is colored on the rainbow scale from the N-terminus (blue) to the C-terminus (red). **B** Two chains of the full-length human (P)RR (residues 17–350) in a cartoon representation. Each chain is colored in rainbow format, except yellow, which indicates TM regions. **C** Surface representation of the full-length human (P)RR homodimer with the two chains colored gray and pale cyan. Residues 47–60, 200–213, and 281 are shown in red, blue, and green, respectively. **D** Evolutionary conservation profile of human (P)RR evaluated in terms of local structural environment. The Consurf scores are shown as dots, which are color-coded according to the local structural environment: solvent accessible, blue; solvent inaccessible, red; dimer interface, green. **E** Regionwise averaged Consurf scores of human (P)RR. The region “others” contains all ECD residues except 47–60, 105–153, 200–213, and 270–296. **F** (P)RR homodimer viewed perpendicular to the twofold axis. One chain is drawn in blue and the other in cyan. Residues 105–153 are colored red. The dimer interface residues are represented by stick models (green). **G** A close-up of solvent-accessible highly conserved residues (green) in two regions: residues 47–60 (red) and 200–213 (blue)

To examine (P)RR homodimerization in silico, AF2 prediction was performed to evaluate the dimerization capability of the two IDR-truncated ECD chains (residues 17–270). The predicted models adopted a homodimeric structure with a high average pLDDT score and low interchain PAE value (Supplementary Fig. 2A). The two chains of ECD assembled to form a twofold symmetric dimer (back-to-back) stabilized through intermolecular interactions between the conserved flat surfaces (left, Fig. 5A). The dimer was composed of one β-sheet united by an edge β-strand from each chain (right, Fig. 5A).

Next, we predicted the homodimeric structure of the full-length human (P)RR (residues 17–350). The intrachain PAE plots indicated that all the models comprised two independent domains in one chain and that the model with the highest pLDDT score exhibited a defined interchain relative position and orientation (Supplementary Fig. 2B). Two chains of full-length human (P)RR formed a back-to-back homodimer via ECD (Fig. 5B). The ProSA Z score of −6.2 for the structural model (Supplementary Fig. 2C) indicated that the model was situated in the region of structures obtained by X-ray crystallography. The Ramachandran plot (Supplementary Fig. 2D) showed that most residues in the folded domain of the ECD occupied the most favored regions. The validation analyses based on the ProSA Z score and Ramachandran plot indicated that our (P)RR structural model was acceptable for use for further analysis in this study.

The twofold symmetric dimer of (P)RR had two surface regions, comprising residues 47–60 and 200–213 of each (P)RR (Fig. 5C and Supplementary Movie 1), that were not buried upon homodimerization (Fig. 5C). Notably, the IDR (residues 270–296) of one chain protruded over residues 47–60 of the other chain (Fig. 5C), forming a flexible flap over the region.

### Conservation profile evaluated with respect to the local structural environment

We first identified solvent-accessible/inaccessible residues and dimer interface residues using the PISA server [37]. A surface area equivalent to 4.5% (1017 Å^2^) of one chain was buried upon homodimerization. Next, per-residue conservation scores were examined based on solvent accessibility and involvement in the dimer interface (Fig. 5D). Notably, very few residues with scores < 5 were observed in the 105–153 region, and approximately half the residues in this region were located at the dimer interface (Fig. 5D). The average conservation score of 6.45 in the 105–153 region was higher than that of the full-length protein (Fig. 5E). When residues 105–153 were mapped to the predicted homodimeric structural model, these conserved residues were found to contribute directly to homodimer formation (Fig. 5F).

The average conservation scores of the 47–60 and 200–213 residue regions were 6.07 and 5.93, respectively. The solvent-accessible and highly conserved (score > 8) residues in the regions containing residues 47–60 and 200–213 were L48, M50, G51, F52, S53, L58, S59, H202, S208, P209, and D210 (Fig. 5D). These residues were arranged in a line on a continuous surface patch (Fig. 5G), which is particularly important in these regions.

**Fig. 6:**
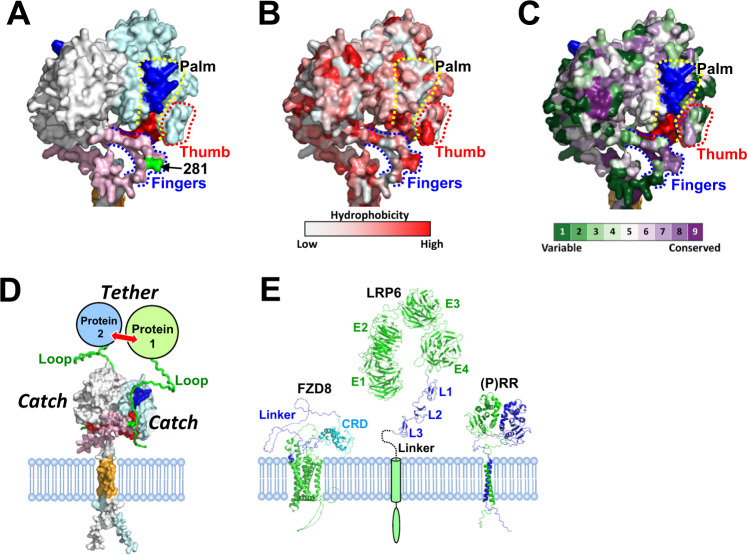
Mapping the protein binding sites of (P)RR. **A** Surface representation of the ECD of the full-length human (P)RR homodimer. Palm, residues 47–60 (blue) and 200–213 (red); thumb, residues 65–70 (red dotted); and fingers, residues 270–296 (blue botted). The model is shown with the same color coding as in Fig. 5C, except the IDR is shown in pink (residues 270–296). Surface representations with hydrophobicity score (**B**) and conservation profile (**C**) are shown in the same orientation as in (**A**). **D** Proposed “catch and tether” mechanism of (P)RR. (P)RR catches a loop with its “hand” and tethers two proteins. **E** AF2 structural models of FZD8, LRP6, and (P)RR depicted on the same scale for comparison. CRD cysteine-rich domain, E1–E4 four β-propeller/epidermal growth factor repeats, L1–L3 three low-density lipoprotein receptor type A repeats. The LRP6 structure downstream from L3 is shown in cartoon representation

Further inspection of the (P)RR structural model revealed the presence of a groove formed by a right-hand-shaped structure with three distinct areas: palm, thumb, and fingers (Fig. 6A). The palm comprised the 47–60 and 200–213 regions. The fingers corresponded to the IDR-based flexible flap mentioned above, and the groove had space to accommodate a single extended loop (Supplementary Fig. 3 and Supplementary Movie 2); the grooves were largely hydrophobic (Fig. 6B). The conservation profile indicated that the palm, thumb, and finger areas consisted of highly conserved residues (Fig. 6C), which implied the functional importance of the “(P)RR hand.”

Based on the common features of the IDR, we anticipated two possibilities for the interaction. In the first, the “(P)RR hand” *catches* the sticky or relatively hydrophobic loop (Supplementary Fig. 3 and Supplementary Movie 2) as a result of the conformational flexibility of the IDR-based fingers. In the second, (P)RR IDR directly *catches* its partner via conformational plasticity during binding. Thus, it is conceivable that the hand-shaped architecture allows (P)RR to *catch* two binding partners and *tether* them closely in space to facilitate protein–protein interactions (Fig. 6D). The hetero-oligomer consisting of two (P)RR and one or two binding partners can be formed adjacent to the cell membrane (Fig. 6D).

## Discussion

A previous in silico analysis by Sanchez-Guerrero et al. [22] generated a 3D structural model of full-length (P)RR and reported the probable binding site residues for two prorenin regions, which have since been shown to be important for (P)RR binding [49]. The constructed structure is monomeric [22]. Here, we revealed new structural features of the binding site of (P)RR. First, the structural basis for homodimerization was determined. Second, the dimeric 3D model demonstrated that two regions involved in the PDAC antiproliferative effect and (P)RR IDR formed two evolutionarily conserved grooves, which probably act as a protein binding site to exert multiple functions, including Wnt signaling activation. This is the first report of (P)RR homodimerization and its multiple functionalities based on 3D structural information. It is notable that L48, M50, G51, F52, and S59 are the protein binding site residues commonly identified in both the previous study [22] and our study (Fig. 5G), although the assembly state and overall 3D structure are not the same in both studies.

Our in silico analysis demonstrated that full-length human (P)RR formed a back-to-back homodimer via the ECD (Fig. 5B). The conservation profile obtained independently of AF2 indicated that residues 105–153 were highly conserved (Fig. 5D, E). The direct contribution of these residues to homodimer formation (Fig. 5F) illustrates the structural importance of these conserved residues and is consistent with the experimentally proven homodimerization [8, 44, 45]. Heterodimer formation between full-length (P)RR and s(P)RR [45] can be achieved through intermolecular interactions between the two ECD surfaces (Fig. 5F).

Upon binding to Wnt ligands, the Frizzled and LRP6 receptors are bridged and assembled into multiprotein complexes termed Wnt signalosomes [50–52]. (P)RR interacts with FZD8 and LRP6 in the extracellular space in a Wnt-independent manner [13]. FZD8 is composed of a cysteine-rich domain (CRD), a flexible and largely unstructured CRD-to-TM linker, and a seven-span TM domain [51] (Fig. 6E). The ectodomain of LRP6 is composed of four β-propeller/epidermal growth factor repeats (E1–E4), three low-density lipoprotein receptor type A repeats (L1–L3), and a short linker [52] (Fig. 6E). Considering the spatial proximity of these proteins to the membrane (Fig. 6E), (P)RR probably interacts with FZD8 ECD (CRD and/or linker) and a part of the LRP6 ectodomain (L1–L3 and/or linker) via the *catch* and *tether* mechanism. This tethering likely facilitates Wnt signalosome formation and would be advantageous for effectively responding to Wnt ligands. pAbs against the 47–60 and 200–213 regions [15] probably interfere with signalosome assembly at the protein-binding interface.

When an interaction partner is supposed to bind with the “(P)RR hand,” its anticipated interaction primarily relies on hydrophobic interaction, which has relatively low stereospecificity and is tolerant to multiple binding orientations. Alternatively, an interaction partner may directly bind to the (P)RR IDR. This binding mode may explain the known one-to-many binding and the multiple functions of (P)RR. Deletion analysis showed that the (P)RR ECD is required for the biogenesis of active V-ATPase [53]. (P)RR binds (directly or indirectly) to the V-ATPase subunits ATP6V0C and ATP6V0D1, where binding of the ATP6V0C subunit is achieved via (P)RR ECD [13]. To facilitate active V-ATPase biogenesis, the multiple subunits consisting of V-ATPase assembly may be *caught* and *tethered* by the “(P)RR hand.” Thus, (P)RR probably functions as an extracellular scaffold protein in Wnt/β-catenin signaling, a V-ATPase assembly factor [54], and a hub in protein–protein interaction networks in various signaling pathways. Abbas et al. [20]. reported that some full-length (P)RRs are present in cryo-EM protein preparations. The cryo-EM structure of a minor population of V-ATPase or Vo complexes that contain intact (P)RR may clarify how the multiple subunits are assembled into V-ATPase with the help of full-length (P)RR.

s(P)RR is considered a useful biomarker for diseases and a biologically active paracrine factor [2]. Site-1 protease (S1P) is responsible for s(P)RR generation, and L281 is the C-terminal residue after S1P cleavage [55]. Because s(P)RR probably retains the hand-shaped architecture (Fig. 6A), it may confer *catch* and *tether* functionality.

The role of (P)RR in blood pressure regulation and the development of hypertension has been unveiled based on (P)RR levels in the brain and kidney [2]. (P)RR protein levels are elevated in the subfornical organ of the brain of hypertensive humans [56], and brain (P)RR can regulate blood pressure by altering prohypertensive and antihypertensive pathways through local angiotensin-II-dependent and angiotensin-II-independent mechanisms [2]. Adrenal gland (P)RR is anticipated to contribute to adrenal aldosterone synthesis and the pathogenesis of hypertension [2, 57], although the precise mechanism leading to the enhancement of aldosterone production remains unknown. In this study, we proposed that the IDR-containing hydrophobic grooves act as a protein binding site of (P)RR and allow multiple protein interactions. Therefore, this study may provide clues to help find new binding proteins for (P)RR and clarify the (P)RR-mediated molecular mechanisms leading to hypertension.

In conclusion, our in silico structural analysis mapped the binding site of (P)RR. This study provides the first 3D structural insight into receptor binding and one-to-many interactions, underpinning the functional versatility of this receptor. Our findings will increase the understanding of disease pathogenesis and explore novel modalities to treat human diseases, including hypertension and cancer. Further analyses are required to experimentally characterize IDR-based protein interactions between (P)RR and its interaction partners.

## Supplementary information

Supplementary Information

Supplementary Movie 1

Supplementary Movie 2
