# Publisher Correction: Mapping the protein binding site of the (pro)renin receptor using in silico 3D structural analysis

**DOI:** 10.1038/s41440-022-01139-0

**Published:** 2022-12-19

**Authors:** Akio Ebihara, Daiki Sugihara, Makoto Matsuyama, Chiharu Suzuki-Nakagawa, A. H. M. Nurun Nabi, Tsutomu Nakagawa, Akira Nishiyama, Fumiaki Suzuki

**Affiliations:** 1 Faculty of Applied Biological Sciences, Gifu University, Tokai National Higher Education and Research System, 1-1 Yanagido, Gifu, 501-1193 Japan; 2 Center for Highly Advanced Integration of Nano and Life Sciences (G-CHAIN), Gifu University, Tokai National Higher Education and Research System, 1-1 Yanagido, Gifu, 501-1193 Japan; 3 Preemptive Food Research Center (PFRC), Gifu University Institute for Advanced Study, 1-1 Yanagido, Gifu, 501-1193 Japan; 4 Department of Chemical Engineering, Indian Institute of Technology Guwahati, Guwahati, Assam 781039 India; 5 Graduate School of Natural Science and Technology, Gifu University, Tokai National Higher Education and Research System, 1-1 Yanagido, Gifu, 501-1193 Japan; 6 Division of Molecular Genetics, Shigei Medical Research Institute, Okayama, Minami 701-0202 Japan; 7 Laboratory of Population Genetics, Department of Biochemistry and Molecular Biology, University of Dhaka, Dhaka, 1000 Bangladesh; 8 Department of Pharmacology, Faculty of Medicine, Kagawa University, Miki, Kagawa 761-0793 Japan

Correction to: *Hypertension Research* 10.1038/s41440-022-01094-w, published online 09 December 2022

Graphical Abstract was missing from this article; the figure should have appeared as shown below.



The original article has been corrected.

