# Supplementary material for: Mapping the protein binding site of the (pro)renin receptor using in silico 3D structural analysis
Source: Hypertens Res. 2022 Dec 9;46(4):959–71. doi: 10.1038/s41440-022-01094-w (PMC10073018; doi:10.1038/s41440-022-01094-w)
Supplement: Supplementary file 1 — Supplementary Information [file 41440_2022_1094_MOESM1_ESM.docx]

**Supplementary Information**

**Mapping the protein binding site of the (pro)renin receptor using in silico 3D structural analysis**

Akio Ebihara^1,2,3,4^, Daiki Sugihara^5^, Makoto Matsuyama^6^, Chiharu Suzuki-Nakagawa^1^, A.H.M. Nurun Nabi^7^, Tsutomu Nakagawa^1^, Akira Nishiyama^8^, and Fumiaki Suzuki^1^

^1^Faculty of Applied Biological Sciences, Gifu University, Tokai National Higher Education and Research System, 1-1 Yanagido, Gifu 501-1193, Japan

^2^Center for Highly Advanced Integration of Nano and Life Sciences (G-CHAIN), Gifu University, Tokai National Higher Education and Research System, 1-1 Yanagido, Gifu 501-1193, Japan ^3^Preemptive Food Research Center (PFRC), Gifu University Institute for Advanced Study, 1-1 Yanagido, Gifu 501-1193, Japan

^4^Department of Chemical Engineering, Indian Institute of Technology Guwahati, Guwahati, Assam 781039, India

^5^Graduate School of Natural Science and Technology, Gifu University, Tokai National Higher Education and Research System, 1-1 Yanagido, Gifu 501-1193, Japan

^6^Division of Molecular Genetics, Shigei Medical Research Institute, Minami, Okayama 701-0202, Japan

^7^Laboratory of Population Genetics, Department of Biochemistry and Molecular Biology, University of Dhaka, Dhaka-1000, Bangladesh

^8^Department of Pharmacology, Faculty of Medicine, Kagawa University, Miki, Kagawa 761-0793, Japan

**Corresponding author:** Akio Ebihara

**Contents**

**Supplementary Table 1.** Results of the Dali search for structures similar to the human (pro)renin receptor [(P)RR].

**Supplementary Fig. 1.** Structural validation results of the AlphaFold structural model of human (P)RR.

**Supplementary Fig. 2.** Analysis of human (P)RR dimer formation and structural validation of the AlphaFold structural model of the full-length (P)RR dimer.

**Supplementary Fig. 3.** Model for loop binding to the (P)RR groove.

**Captions for Supplementary Movies 1 and 2 (see associated mp4 files)**

The two chains are colored gray and pale cyan. Residues 47–60, 200–213, and 281 are indicated by red, blue, and green, respectively. In **Supplementary Movie 2**, the intrinsically disordered region (residues 270–296) is pink, and the green tube denotes part of the Frizzled-8 linker fitted to the groove of the extracellular domain (ECD).

**Supplementary Movie 1.** Surface representation of the predicted dimeric structure of the full-length (P)RR is shown in **Fig. 5C**.

**Supplementary Movie 2.** Surface representation of the predicted dimeric structure of the full-length (P)RR is shown in **Supplementary Fig. 3**.

**Supplementary Table 1. Results of Dali search for structures similar to human (P)RR.**

| PDB code | Protein description | Z-score | RMSD (Å) | Aligned residues | Number of residues | Sequence identity (%) | Species | Molecular function |
| --- | --- | --- | --- | --- | --- | --- | --- | --- |
| 5XWK | Alkaline phosphatase PhoK | 10.3 | 3.7 | 192 | 530 | 14 | *Sphingomonas sp* | GO:0004035: alkaline phosphatase activity |
| 3IGY | Cofactor-independent phosphoglycerate mutase | 7.5 | 3.2 | 149 | 549 | 8 | *Leishmania mexicana* | GO:0004619: phosphoglycerate mutase activity |
| 6XLP | Lipopolysaccharide-binding protein | 7.3 | 8.8 | 161 | 586 | 12 | *Escherichia coli* | GO:0008484: sulfuric ester hydrolase activity |
| 6C01 | Ectonucleotide pyrophosphatase/phosphodiesterase 3 (ENPP3) | 7.0 | 4.1 | 171 | 819 | 9 | *Homo sapiens* | GO:0004528: phosphodiesterase I activity |
| 4MIV | *N*-Sulfoglucosamine sulfohydrolase | 6.1 | 4.9 | 162 | 480 | 7 | *Homo sapiens* | GO:0008484: sulfuric ester hydrolase activity |
| 5FQL | Iduronate-2-sulfatase | 6.1 | 4.3 | 162 | 507 | 7 | *Homo sapiens* | GO:0004423: iduronate-2-sulfatase activity |
| 4KJG | Intestinal-type alkaline phosphatase 1 | 6.0 | 6.2 | 150 | 486 | 10 | *Rattus norvegicus* | GO:0004035: alkaline phosphatase activity |
| 1P49 | Steryl-sulfatase | 6.0 | 4.0 | 161 | 549 | 7 | *Homo sapiens* | GO:0008484: sulfuric ester hydrolase activity |
| 4CGZ | Bloom's syndrome helicase | 3.0 | 6.9 | 124 | 629 | 6 | *Homo sapiens* | GO:0004386: helicase activity |

PDB, Protein Data Bank; RMSD, root mean square deviation


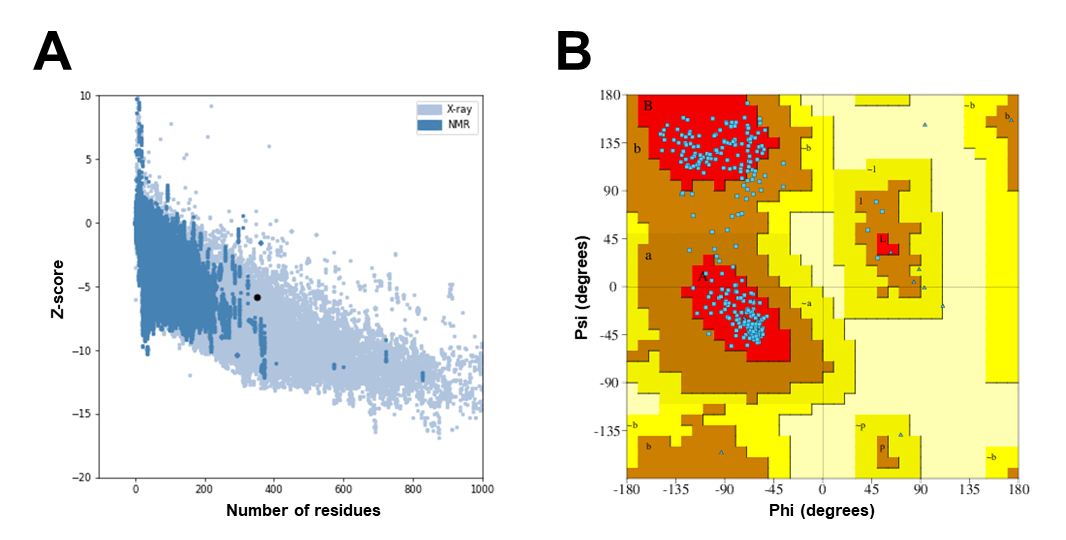


**Supplementary Fig. 1**. Structural validation results of the AlphaFold structural model of human (P)RR. (A) ProSA plot data. The overall quality of the protein structure can be evaluated from the plot, in which the Z-scores of all protein chains determined by X-ray crystallography (light blue) or NMR spectroscopy (dark blue) are plotted with respect to their length. The black dot corresponds to human (P)RR with a Z-score of −5.83. (B) Ramachandran plot (UniProt accession ID: O75787) data. The most favored and additional allowed regions are colored in red and brown, respectively. The generously allowed and disallowed regions are yellow and pale yellow, respectively.


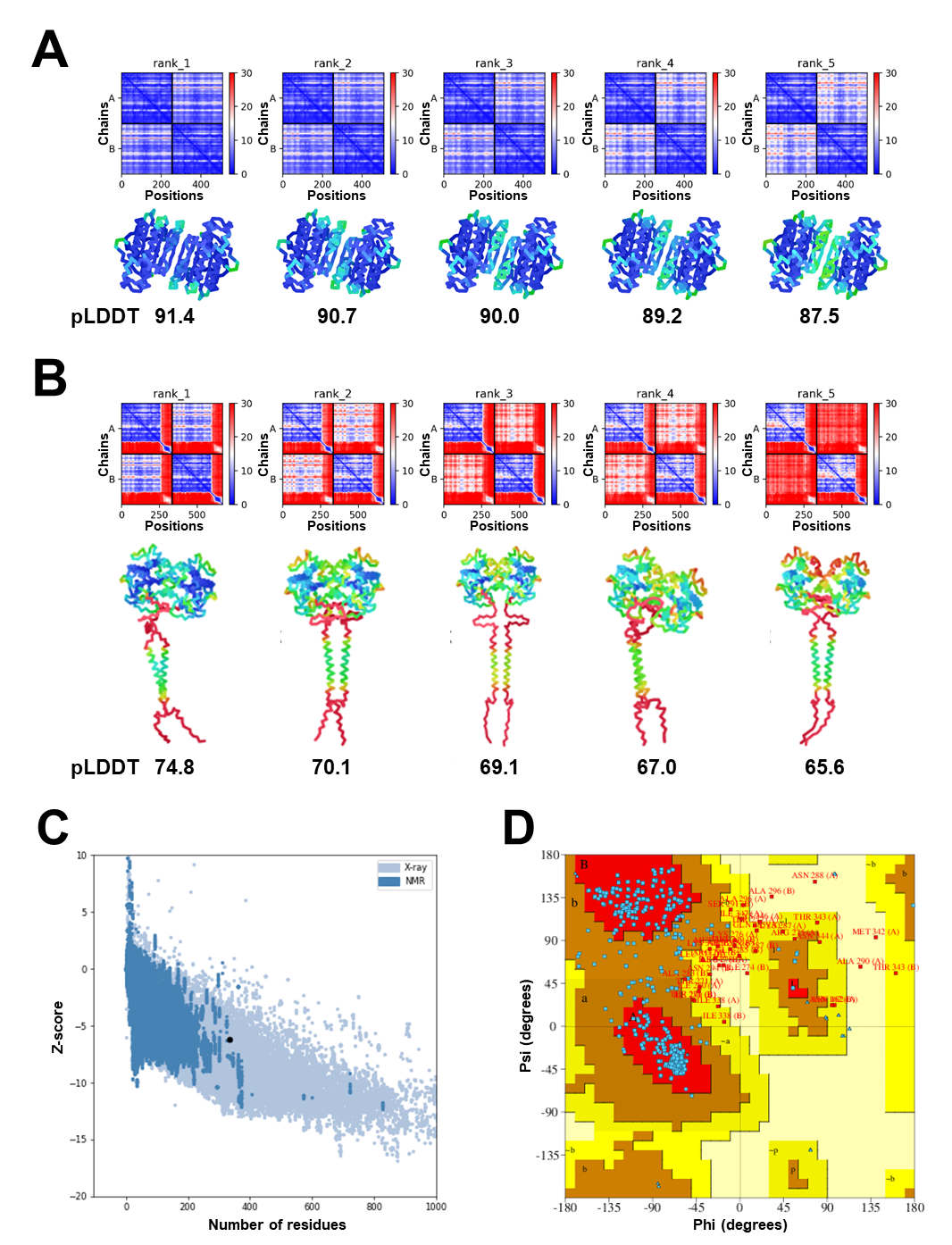


**Supplementary Fig. 2**. Analysis of human (P)RR dimer formation and structural validation of the AlphaFold structural model of the full-length (P)RR dimer. (A) Two chains of human (P)RR ECD (residues 17–270) and (B) two chains of full-length human (P)RR (residues 17–350). Each panel contains the predicted aligned error (PAE) plots of the resulting five models (top) and their structural models (bottom). PAE color-coding bar is shown right to the plot. PAE plots of panels A and B were generated for 254 and 334 residues of each chain (i.e., chains A and B), respectively. In panels A and B, five models are ranked by their average predicted local-distance difference test (pLDDT) scores (shown below each model). The models are colored according to the pLDDT score. (C) ProSA plot of the predicted dimeric structure of full-length human (P)RR. The black dot corresponds to the human (P)RR dimer having a Z-score of −6.2. (D) Ramachandran plot of human (P)RR dimer. The plot indicates that 81.5% of the residues were in the most favored regions, 12.3% in the additional allowed regions, 5.0% in the generously allowed regions, and 1.2% in the disallowed regions


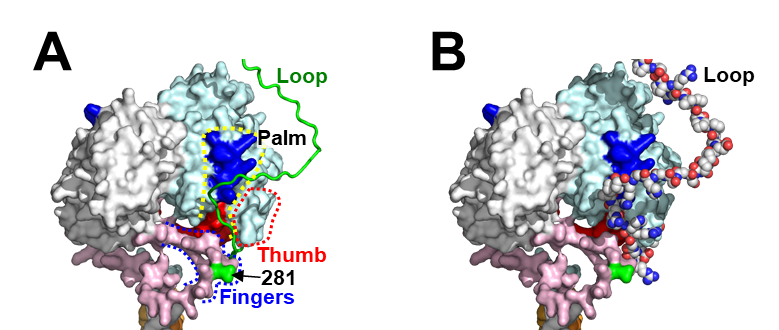


**Supplementary Fig. 3**. Model for loop binding to the (P)RR groove. A part of Frizzled-8 linker (residues 156–206; AF-Q9H461-F1) was used as an example of loop structure and manually fitted to the groove formed by the palm, thumb, and fingers areas. The loop is shown in green tube (A) and sphere representation (B) with carbon in gray, with nitrogen in blue and oxygen in red.
